# Segmentation and structural connectivity of the putamen for targeted convection enhanced drug delivery in Parkinson’s disease: a tractography-based approach

**DOI:** 10.3389/fnins.2026.1598062

**Published:** 2026-02-23

**Authors:** Edgar Tessmann, Marianne Schell, Sandro M. Krieg, Martin Jakobs

**Affiliations:** 1 Department of Neurosurgery, Heidelberg University Hospital, Heidelberg, Germany; 2 Medical Faculty, Heidelberg University, Heidelberg, Germany; 3 Division for Stereotactic Neurosurgery, Department of Neurosurgery, Heidelberg University Hospital, Heidelberg, Germany; 4 Department of Neuroradiology, Heidelberg University Hospital, Heidelberg, Germany

**Keywords:** convection-enhanced drug delivery (CED), diffusion-weighted imaging (DWI), parcellation, Parkinson’s disease (PD), structural connectivity, tractography

## Abstract

**Background:**

Convection-enhanced drug delivery (CED) for Parkinson’s disease (PD) is costly, and current methods lack precision, often targeting the entire putamen, leading to inefficient use of resources. Our simulation study explores a more targeted approach, which could reduce treatment costs by focusing therapy on specific putaminal regions, aiming to optimize delivery without compromising efficacy.

**Methods:**

Twenty PD patients underwent diffusion-weighted imaging (DWI) to visualize the structural connectivity. A commercial subcortical auto-segmentation tool was used to define the putamen as well as the amygdala, the STN, and the cerebellum. Utilizing the Julich Brain Atlas, nine cortical regions (Brodmann areas 44, 45, 3a/b, 4a/p, pre-SMA, SMA, and insula) were semi-automatically segmented. Before tractography, the putamen was pre-parcellated into four segments in relation to the anterior commissure. Tractography was then performed to assess the cortical connectivity of each segment. We evaluated two virtual injection trajectories (occipital and frontal) and simulated stepwise infusions of a therapeutic agent. A genetic algorithm optimized the simulated infusions and compared coverage of the target region.

**Results:**

Tractography revealed a significant projection of motor areas to the superior posterior segment of the putamen, suggesting this region as a more specific target for treating motor symptoms in PD via CED. Non-motor connections were most common in the inferior posterior segment for the amygdala and in the superior anterior segment for the insula. Both occipital and frontal trajectories were found to be equally feasible for targeting the putamen segments, with surgical feasibility varying by individual patient anatomy, and achieved comparable coverage, with no significant difference between them, highlighting the need for personalized surgical approaches.

**Conclusion:**

DWI-based deterministic tractography mapped motor cortical projections most consistently to the superior-posterior putamen in our AC-based segmentation. In our planning simulations, both frontal and occipital trajectories could be planned with similar safety and achieved similar simulated coverage of the segment. These results support using connectivity mapping and simple simulation as a planning adjunct to help select a motor-dominant putaminal target and compare trajectories. If validated clinically, targeting a motor-dominant segment could reduce exposure of non-motor putaminal regions and potentially reduce the required infusion volume.

## Introduction

Parkinson’s disease (PD) is a progressive neurodegenerative disorder that primarily affects motor function, significantly impairing patients’ quality of life. Current treatment options, while effective in managing symptoms, often become less efficient over time, prompting the need for innovative therapeutic approaches (Hwu et al., 2021). Convection-enhanced drug delivery (CED) has emerged as a promising strategy for targeted drug administration in PD, particularly for cell and gene therapy. CED infuses therapeutic agents via stereotactically placed catheters under positive pressure to drive bulk flow through the interstitium, achieving high local concentrations over larger brain volumes, reducing systemic side effects and improving local drug concentration (Lam et al., 2011; Barua et al., 2014). In practice, distribution is constrained by anatomical variability, tissue anisotropy, and potential reflux along the catheter track, which can challenge precise targeting (Lam et al., 2011; Barua et al., 2013; Larson, 2021).

The putamen, as part of the basal ganglia, plays a key role in motor control and is a central target for CED in PD therapy (Miranpuri et al., 2012). Conventional CED methods, however, often target the entire putamen, resulting in inefficient drug use and unnecessary exposure of non-motor regions. This broad targeting not only increases treatment costs by requiring more of the drug but also side effects by affecting regions that do not contribute to motor function. Thus, more precise drug delivery methods that target specific, motor-relevant areas of the putamen are needed to improve therapeutic efficacy and reduce unnecessary drug use. Prior work using similar structural or multimodal approaches, such as combining resting-state fMRI and diffusion MRI tractography, have delineated putaminal subregions with distinct connectivity (Draganski et al., 2008; Tian et al., 2020). Analogous to the principle of applying deep brain stimulation (DBS) for PD in the dorsolateral motor part of the subthalamic nucleus (STN) to achieve the optimal motor improvement, these findings suggest an opportunity to focus CED therapy on motor-connected segments rather than the entire putamen.

The goal of this study is to use existing connectivity knowledge to explore simulation prior to CED and inform the planning. In a clinical workflow, an easily identifiable predefined parcellation of the putamen is applied, then diffusion-weighted imaging (DWI) tractography maps structural connectivity onto these segments to verify the expected motor bias in the putamen. On this basis, the feasibility of connectivity guided CED is evaluated by using two planned trajectories and an in-house developed stepwise-injection simulation that quantifies target coverage. A genetic algorithm optimizes a theoretical stepwise infusion protocol to increase coverage of the selected segment while respecting safety constraints. The distribution model is kept simple to keep assumptions explicit and computations tractable. Simulation outputs are therefore interpreted as planning aids—to quantify safety, coverage and to support case-specific decisions—rather than predictors of *in vivo* distribution. While clinical validation is not yet available for the present application, MRI-based presurgical planning tools in other neurosurgical settings have been evaluated prospectively and shown to improve perioperative outcomes and functional status compared with conventional planning (Yang et al., 2009). The objective is to assess the clinical feasibility of connectivity-guided CED in the putamen with safety and coverage metrics across the two trajectories.

## Methods

### Patients

This study included the retrospective analysis of MRI scans from 20 adult patients (both male and female) diagnosed with advanced Parkinson’s disease (PD). The MRI scans were part of the preoperative protocol before undergoing DBS surgery for PD, for which all participants were cleared.

### Imaging and processing

Prior to DBS treatment, all patients underwent pre-operative MRI imaging as part of the standard surgical planning process. The MRI protocol included T1, T2, FGATIR, FLAIR, SWI, and DWI sequences. The DWI sequence was a single-shot spin-echo echo-planar imaging (EPI) acquisition with 64 diffusion-encoding directions and a b-value of 1,000 s/mm^2^. Voxel size was 2.0 × 2.0 × 2.0 mm^3^, with TR 5100 ms, TE 64 ms, a 128 × 128 matrix, and a field of view of 256 × 256 mm^2^ in transversal orientation. All remaining acquisition parameters are provided in Supplementary material. After acquisition, raw DICOM images were converted into NIfTI format. Brain extraction was applied to T1 and T2 images, and eddy current correction was performed on the DWI data using the FMRIB Software Library (FSL) version 6.0 (Smith et al., 2004; Woolrich et al., 2009; Jenkinson et al., 2012). For accurate multimodal alignment, all non-T1 sequences were rigidly coregistered to the native T1-weighted image. T1-weighted images were then nonlinearly registered to MNI152 Nonlinear 2009c Symmetric space (Fonov et al., 2011) using FNIRT (Woolrich et al., 2009; Andersson et al., 2010), and the same transformation was applied to all other sequences. Subcortical segmentation and tractography were performed using Brainlab Software (Brainlab, 2024). The cortical regions were downloaded from the Julich-Brain Atlas (v3.1) already in MNI152 Nonlinear 2009c Symmetric space (Amunts et al., 2024). The visualizations were rendered in 3D Slicer (Fedorov et al., 2012) and Lead-DBS (Horn and Kühn, 2015; Treu et al., 2020).

### Cortical and subcortical segmentation

Cortical and subcortical connections from the putamen were chosen after literature research and included motor and non-motor projections. The subcortical structures, such as the putamen, amygdala, subthalamic nucleus (STN), and cerebellum, were automatically segmented by Brainlab Software, as routinely used for DBS planning in clinical practice, and then visually confirmed by the attending neurosurgeon (MJ) with reference to T1, T2, and FGATIR images. Cortical brain regions were segmented in a semi-automated fashion using the Julich-Brain Atlas. For each patient, the warp from patient space to MNI space was calculated using FSL software. The cortical regions of interest, defining Brodmann areas 44, 45, 3a/b, 4a/p, 6ma pre-SMA, 6mp SMA, and insula, as previously used for putamen segmentation and supplemented with additional areas for more granularity (Behrens et al., 2003; Bohanna et al., 2011), were downloaded from the Julich-Brain Atlas and then converted to patient space using the inverted warp. The segmentation masks were then transferred to the Brainlab Software to adapt the masks to the cortical anatomy of the respective patient, hence the “semi-automated” approach. The segmentations were manually adjusted by a resident in neurosurgery (ET) and confirmed by an attending neurosurgeon (MJ), using T1 and T2 images to ensure anatomical accuracy.

### Putamen segmentation

The putamina of each patient were segmented in the patient space into four regions based on its anatomical relationship to the anterior commissure (AC). The putamen was divided into superior anterior, superior posterior, inferior posterior, and inferior anterior segments. The AC-PC line served as the axial plane for distinguishing superior and inferior segments, while the coronal plane perpendicular to the AC-PC line was used to differentiate anterior from posterior segments. This division, albeit resulting in differently sized subsections of the putamen, was chosen for its strong anatomical reproducibility and practicality in clinical stereotactic neurosurgery. More balanced methods, such as volumetric equalization or centroid-based division, were considered but not adopted due to their increased complexity, potential for inconsistency, and limited gain in correlation with functional or structural organization.

### Tractography

Tractography was conducted in the patient space using deterministic, diffusion tensor imaging (DTI)-based streamline tracking, implemented in the Brainlab Elements Fibertracking module. The available software supports single-tensor DTI modelling. This method was used in this study due to its clinical availability in CE-marked (CE marking under EU medical-device regulation) planning software and its established role in everyday neurosurgical practice. While probabilistic tractography with more complex [for example constrained spherical deconvolution (CSD)-based] underlying models may offer advantages in detecting subtle or crossing fibers, it is not routinely accessible for clinical use. The putamen segments served as seed regions, and the aforementioned cortical and subcortical areas served as target regions. If any streamlines were observed between a cortical region and a specific putamen segment, a connection was considered present. The following criteria were used to visualize the fibers: a minimum fractional anisotropy (FA) of 0.2, a minimum fiber length of 20 mm, and a maximum fiber angulation of 50°.

### Trajectories

Following the identification of the putamen segment with the highest connectivity strength of motor-associated cortical connections, frontal and occipital trajectories were planned in the patient space using a commercially available stereotactic software (inomed Medizintechnik, 2010). The target points were chosen to maximize the length of the trajectory within the segment, ensuring optimal coverage while adhering to established safety margins. The trajectories were planned by an attending neurosurgeon specializing in functional neurosurgery.

### Injection simulation

An in-house developed computational algorithm using Python (3.10) (with the following libraries: panda, numpy, nibabel and deap) was employed to simulate drug injections into a specific putamen segment and to optimize the placement of injection sites within the putamen segment using a genetic algorithm. The algorithm operated as follows:

- Image data loading and preprocessing: Co-registered NifTI-format images were loaded using the open-source nibabel package. Brain anatomy and segmentation masks for the putamen were extracted. The voxel dimensions were computed based on the image affine transformation to account for spatial scaling during analysis.
- Trajectory generation: Injection trajectories were computed from the entry point to the target using a linear interpolation method, dividing the trajectory into 100 equidistant steps. The trajectory points that intersected with the target region of interest (ROI) within the putamen were identified and used as valid centers for further calculations.
- Sphere generation and coverage calculation: For each valid center along the trajectory, spherical masks were generated using a voxel-wise Euclidean distance metric to simulate the drug diffusion from the injection site. The mask radii were iteratively optimized based on anatomical constraints to ensure the spheres remained entirely within the boundaries of the predefined brain ROI. The total coverage of the target region by these spheres was calculated as the overlap between the sphere masks and the segmentation mask of the ROI.
- Genetic algorithm optimization: A genetic algorithm (GA) was employed to optimize the configuration of injection spheres with respect to their placement and radius. Individuals in the GA population were represented as lists containing the number of spheres, the radii of each sphere, and the coordinates of their centers. The fitness of each individual was determined based on the fraction of the target ROI covered by the corresponding spheres. The GA was initialized with a random population of individuals, and operations, including crossover and mutation, were applied to evolve the population over 20 generations.

○ Mutation: The mutation operation randomly modified the position of one of the spheres by selecting a new valid center from the trajectory.
○ Crossover: Spheres were swapped between two individuals at random points to create offspring, thus encouraging the exploration of new spatial configurations.
○ Fitness evaluation: The fitness of individuals was evaluated as the coverage of the target region by the spheres normalized by the total volume of the segmentation mask. Spheres that exceeded the anatomical boundaries of the putamen or the ROI were penalized by reducing their effective radius.

- Saving results: After the optimization process, the best-performing individual, representing the optimal injection configuration, was selected. The corresponding mask was saved as a NIfTI image for visualization and further analysis. The volume of each sphere and the overall coverage of the target area were also computed and recorded.

### Data analysis

#### Segmentation and connectivity

Heat maps were generated to visualize the connectivity patterns between cortical and subcortical regions and the segmented putamen. The heatmaps display the number of hemispheres (0–40 from 20 patients) with a present connection (≥1 streamline meeting FA/length/angle thresholds) for each segment-ROI pair. The resulting connectivity matrices were plotted as heat maps using GraphPad Prism 9.0 (GraphPad Software, 2020), where the intensity of each cell represented the strength of connectivity between a specific cortical/subcortical region and a putamen segment.

#### Trajectory feasibility

For the analysis of possible trajectories, each trajectory from the cortex to the target region within the specific putamen segment was modelled based on anatomical constraints. Safety margins around critical structures, such as vasculature and ventricles, were calculated using a margin threshold of 2 mm to ensure the trajectory did not violate any anatomical boundaries. Trajectories were planned and assessed for feasibility by an attending neurosurgeon specializing in functional neurosurgery. To evaluate the feasibility between the frontal and occipital trajectories, a mid-p McNemar’s test was performed on paired binary outcomes (0 = unsafe, 1 = safe) within the same patients. Each of the 20 patients had both frontal and occipital trajectories planned for the left and right putamen, resulting in 40 paired observations for each trajectory type. The mid-p McNemar’s test was chosen due to paired nominal data and the small number of discordant pairs in the dataset. The null hypothesis was that there is no significant difference in safety between the frontal and occipital trajectories. Statistical analyses were performed using GraphPad Prism 9.0, with statistical significance set at *p* < 0.05.

#### Coverage comparison

To assess the efficacy of different injection trajectories, a paired *t*-test was used to compare the coverage between the two trajectory types within the same patients. The null hypothesis tested was that there was no significant difference in the coverage between the frontal and occipital approaches. Statistical significance was set at *p* < 0.05.

## Results

A total of 20 patients were included in the analysis. Fourteen patients (70%) were male and six (30%) were female. The mean age at the time of implantation was 59.3 ± 8.9 years (range 40.5–70.1 years). According to the Hoehn & Yahr scale, 11 patients (55.0%) were classified as stage III, 4 (20.0%) as stage II, 4 (20.0%) as stage IV, 1 (5.0%) as stage I.

### Putamen connectivity heat maps

**Figure 1:**
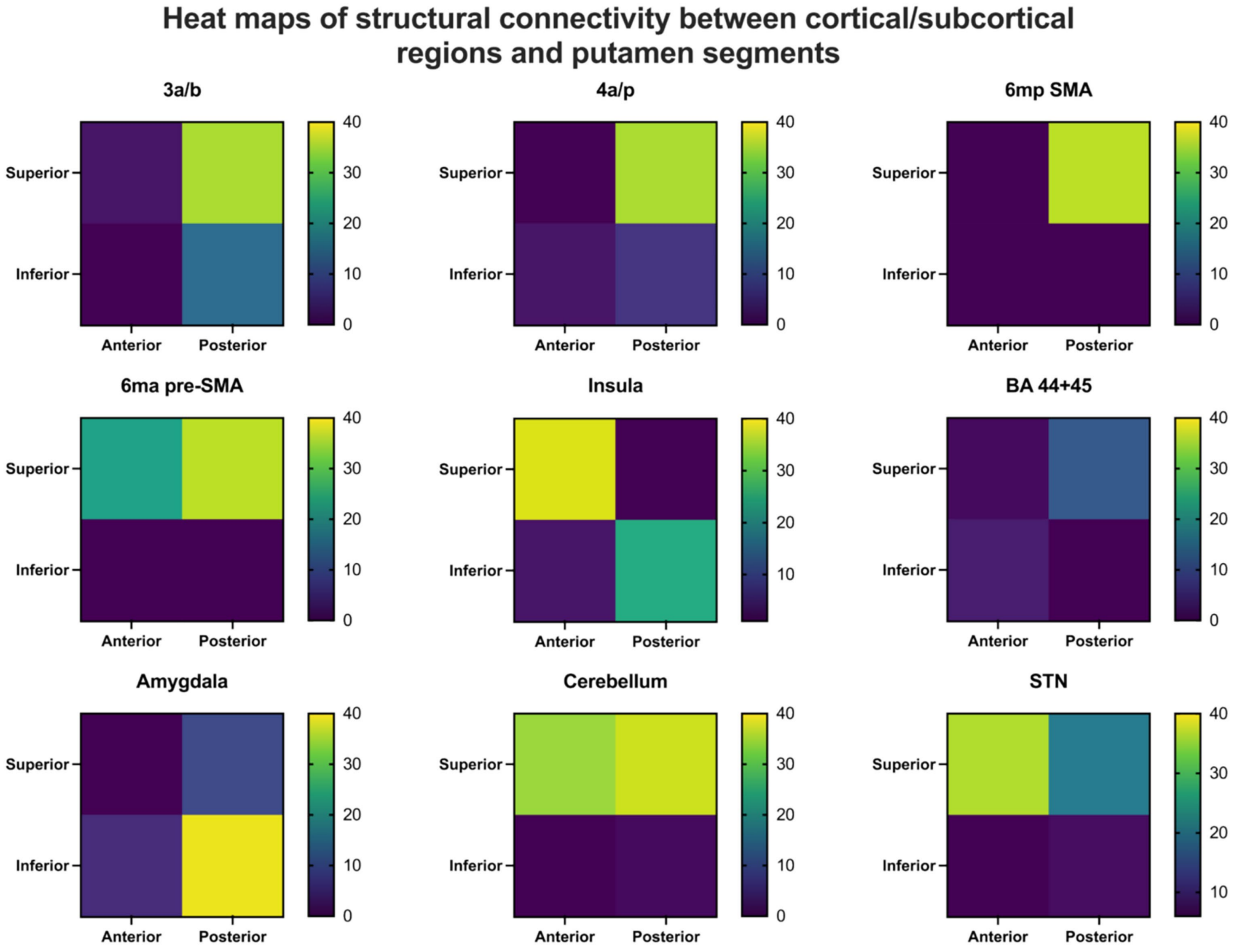
Cohort-level heat maps of connection presence counts of structural connectivity between cortical/subcortical regions and putamen segments. Heat maps show connectivity patterns between motor and non-motor regions and segments of the putamen based on bilateral analysis across 40 hemispheres (20 patients × left/right). Color scale: 0–40 hemispheres with a present connection (≥1 streamline). Motor-associated areas, including regions 3a/b, 4a/p, 6mp SMA, 6ma pre-SMA, cerebellum, and STN, have a high frequency of connections to the superior posterior putamen, with the 6mp SMA connecting almost exclusively to this segment. Non-motor regions reveal different patterns, such as the insula connecting mainly to the superior anterior segment, while the amygdala connects almost entirely to the posterior inferior segment. The superior posterior segment was selected for further analysis due to its connectivity strength to motor-associated cortical and subcortical areas.

The structural connectivity analysis between the selected cortical/subcortical regions and putamen segments demonstrated a distinct pattern for motor-associated areas, including regions 3a/b, 4a/p, 6mp SMA, 6ma pre-SMA, the cerebellum, and the subthalamic nucleus (STN). These regions showed a higher frequency of patients with connections to the superior posterior segment of the putamen. Notably, the 6mp SMA exhibited connections almost exclusively with the posterior superior segment, while other motor-related areas displayed more varied connectivity patterns. For instance, regions such as 3a/b were predominantly connected to posterior regions, whereas the cerebellum and STN were primarily linked to the superior parts of the putamen (Figure 1).

In contrast, non-motor-associated regions revealed distinct connectivity patterns as well. The insula showed high connectivity strength to the superior anterior segment, while the amygdala was almost exclusively connected to the posterior inferior segment of the putamen.

**Figure 2:**
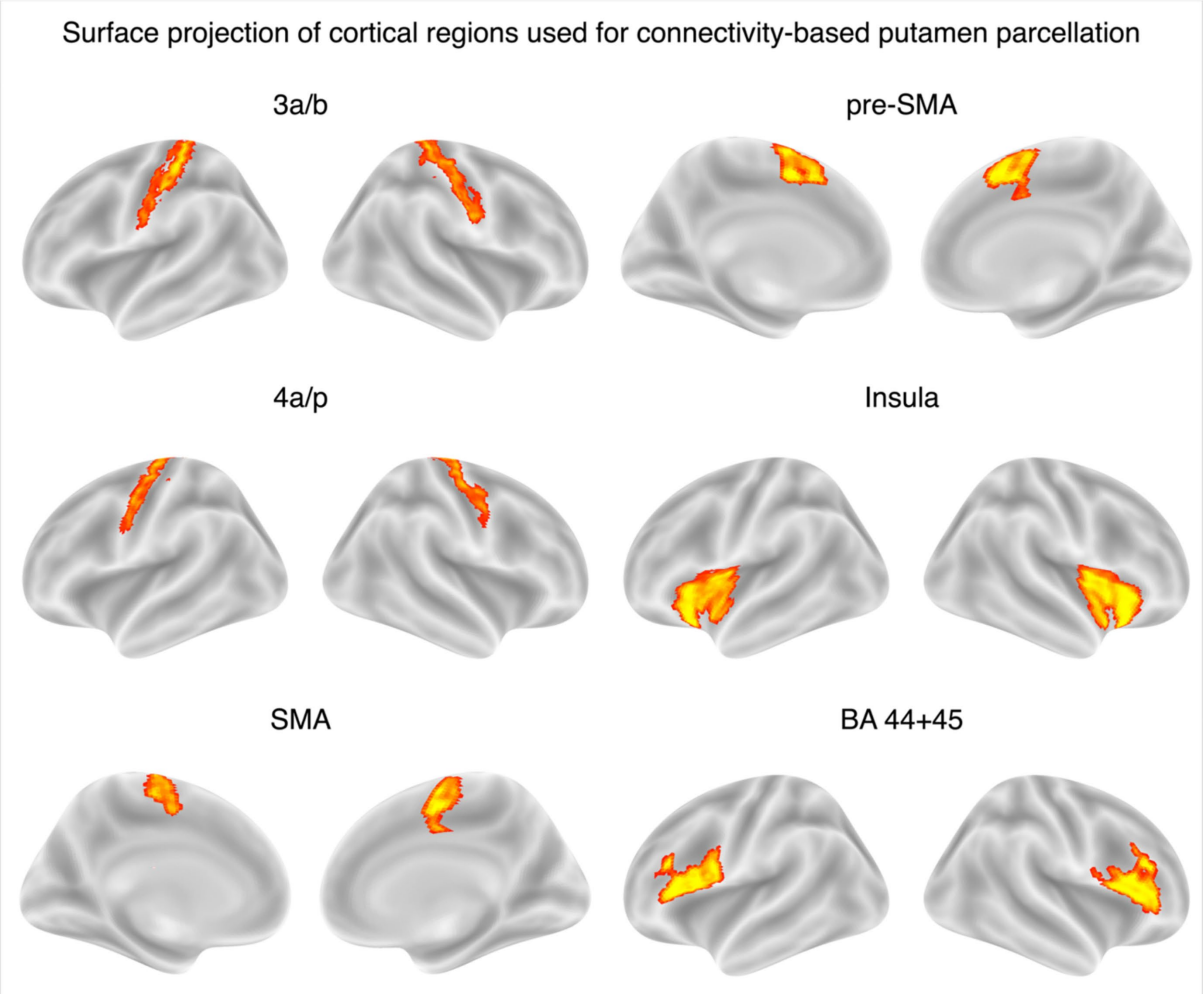
Surface projection of cortical regions used for connectivity-based putamen parcellation. Lateral and medial surface views of cortical regions selected for structural connectivity analysis. Each cortical area is displayed with its corresponding projection in standard MNI space, shown separately for left and right hemispheres and arranged by hemisphere and view. Only medial views are shown for regions involving the supplementary motor area (SMA and pre-SMA). All regions were defined using the Julich-Brain Atlas and registered to patient space via inverse warping for further analyses.

A surface projection of the cortical regions included in the connectivity analysis is shown in Figure 2, providing an overview of the cortical areas used in the analysis.

For subsequent analyses, the superior posterior segment of the putamen was selected due to its high connectivity strength with motor-associated regions.

### Feasibility of frontal and occipital trajectories

**Figure 3:**
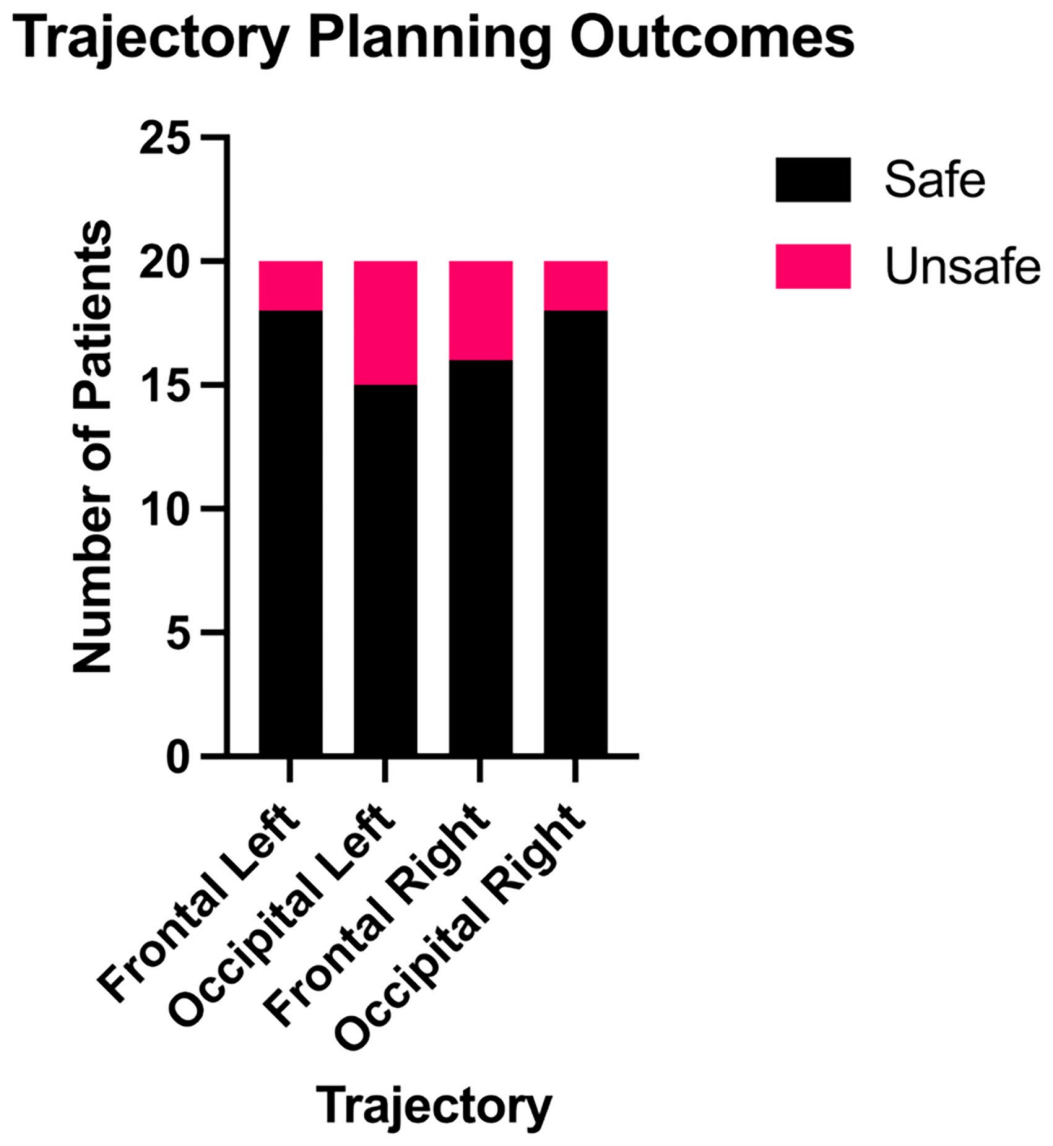
Trajectory planning outcomes by trajectory type. Stacked bar chart displaying safe and unsafe outcomes for each trajectory type: frontal left, occipital left, frontal right, and occipital right. Analysis with a mid-p McNemar’s test revealed no significant difference in feasibility between frontal and occipital approaches, with at least one safe trajectory possible in each patient. However, one patient presented anatomical limitations that prevented the safe planning of any frontal trajectories.

**Figure 4:**
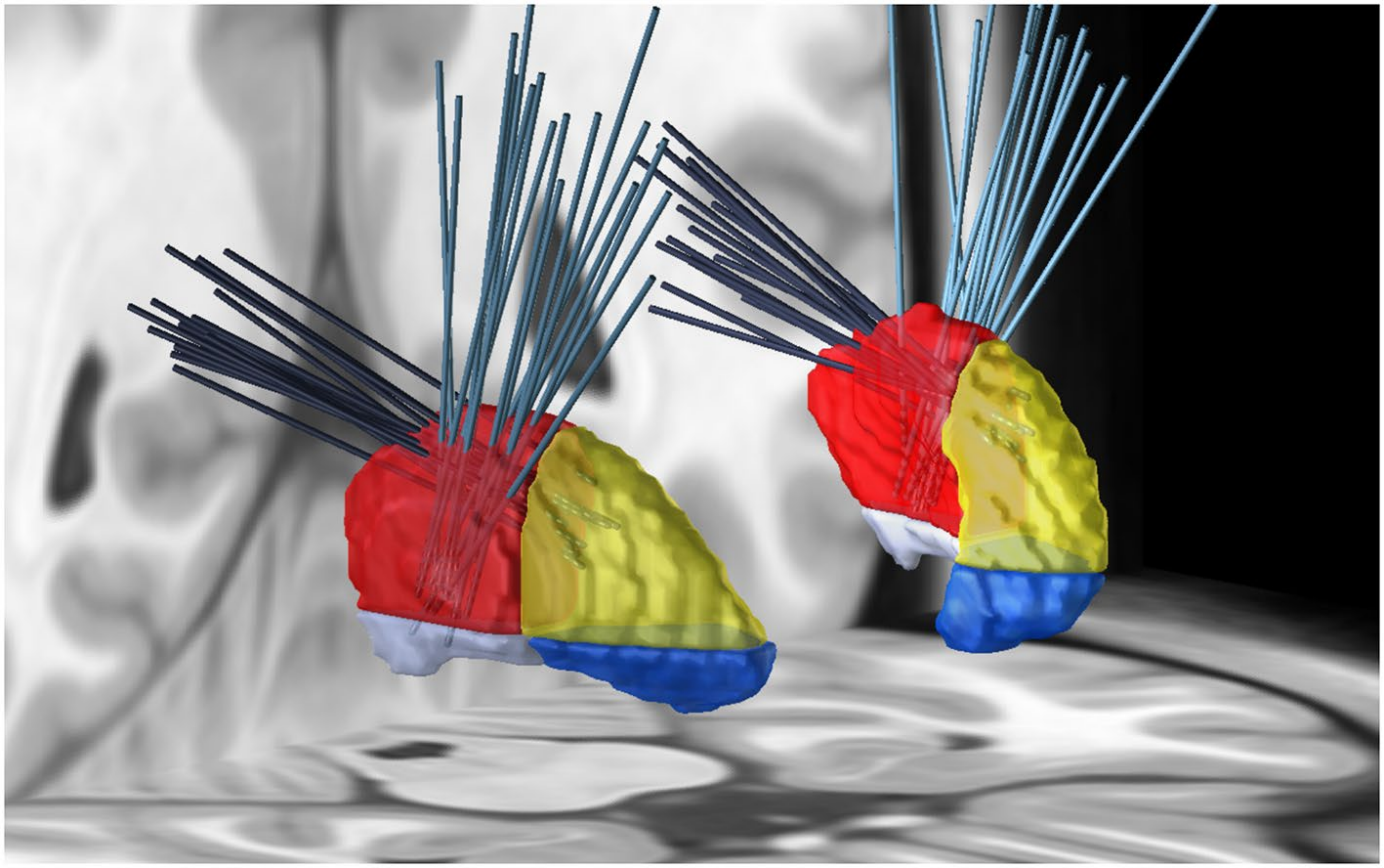
Visualization of all planned drug injection trajectories in MNI space using the Lead DBS software. Frontal trajectories are represented in petrol, and occipital trajectories in dark grey. The putamen is segmented into regions based on relation to the AC and AC-PC line: superior and inferior segments are distinguished along the axial plane aligned with the AC-PC line, while anterior and posterior segments are separated by the coronal plane perpendicular to the AC-PC line. The segmentation includes four regions: superior posterior (red), superior anterior (yellow), inferior anterior (blue), and inferior posterior (grey).

Analysis of the frontal and occipital trajectories revealed no significant difference in the feasibility of either approach (Figure 3). Across all patients, no anatomical constraints were identified that would prevent planning at least one trajectory per hemisphere while adhering to the required safety margins (Figure 4). However, in one patient, it was not possible to plan any frontal trajectories safely due to anatomical limitations and pronounced atrophy.

To statistically assess the difference in feasibility between the frontal and occipital trajectories, a mid-p McNemar’s test was performed on the paired binary outcomes (safe vs. unsafe) for each trajectory type (Table 1).

**Table 1:** Contingency table for safety outcomes of frontal and occipital trajectories.

	Occipital safe	Occipital unsafe	Total
Frontal safe	*a* = 27	*b* = 7	34
Frontal unsafe	*c* = 6	*d* = 0	6
Total	33	7	40

The proportion of safe trajectories was 85% (34 out of 40) for the frontal approach and 82.5% (33 out of 40) for the occipital approach. The total amount of discordant cases (b and c) was 13 pairs. Specifically, 53.8% (7 out of 13) of the discordant pairs favored the frontal trajectory being safe, while 46.2% (6 out of 13) favored the occipital trajectory being safe. The mid-p McNemar’s test yielded a *p*-value of 0.8462, indicating no statistically significant difference in safety between the frontal and occipital trajectories.

### Efficacy of trajectories

**Figure 5:**
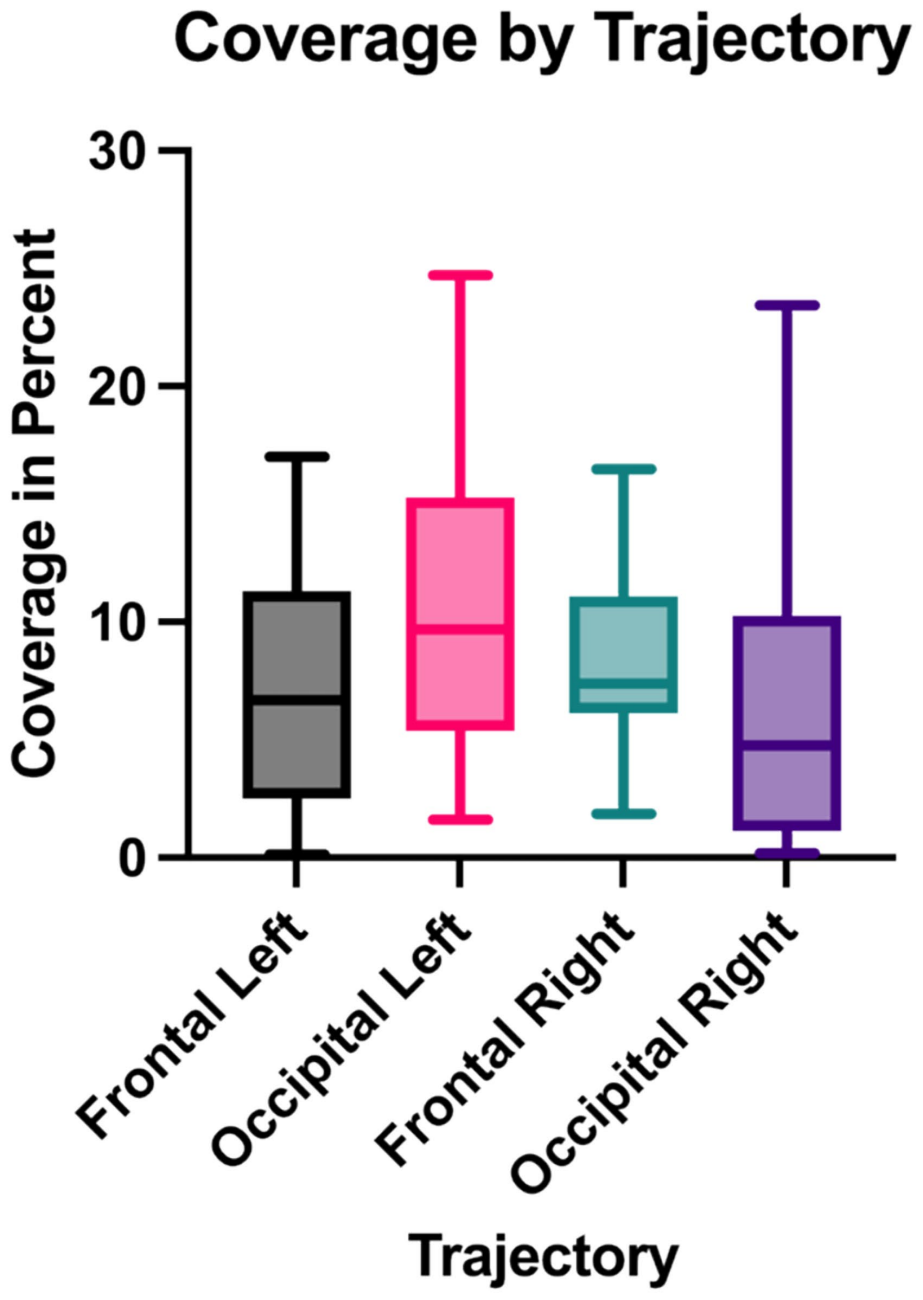
Distribution of coverage by trajectory type. Box plots showing the distribution of coverage percentages for frontal and occipital trajectories, with individual data points paired for each patient (paired *t*-test). On average, frontal trajectories achieved 7.69% coverage (95% CI: 6.17–9.21%), while occipital trajectories achieved 8.74% (95% CI: 6.35–11.14%). The mean difference in coverage between the two approaches was 1.06% (95% CI: −1.99 to 4.11%), with no significant difference detected (*p* = 0.4875).

**Figure 6:**
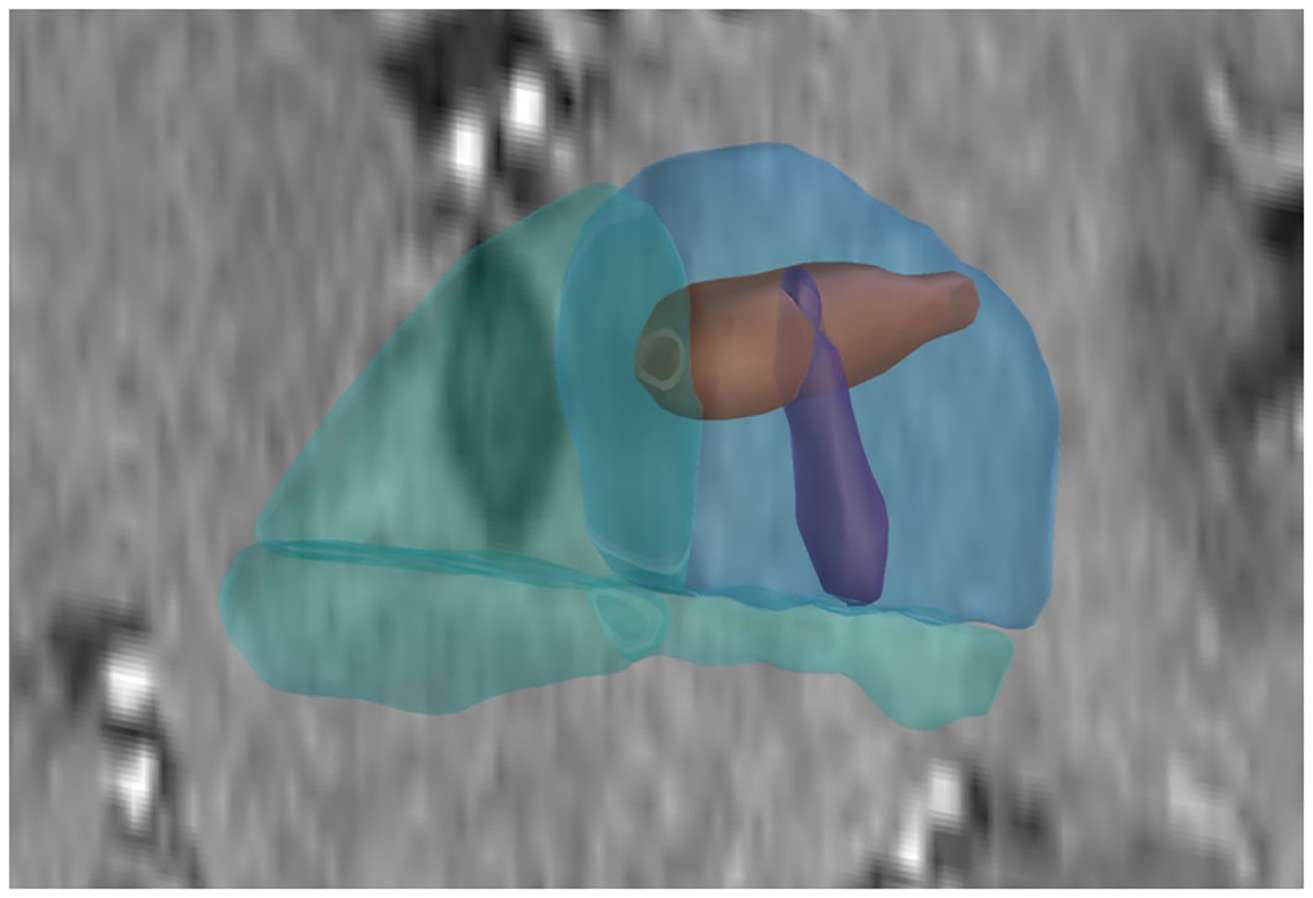
Example of a simulated injection volume in 3D Slicer. The light blue region represents the target segment (superior posterior putamen), while the remaining segments are shown in light green. Simulated injection volumes are depicted in red for the occipital and purple for the frontal trajectory.

Further analysis of coverage within the same patients, using a paired t-test, showed no significant differences between the frontal and occipital trajectories. On average, the percentage coverage of the selected putamen segment was 7.69% (95% CI: 6.17–9.21%) for the frontal trajectory and 8.74% (95% CI: 6.35–11.14%) for the occipital trajectory. The mean difference in coverage between the two approaches was 1.06% (95% CI: −1.99 to 4.11%), with a p-value of 0.4875 (Figures 5, 6).

## Discussion

We aimed to characterize how motor- and non-motor-associated structural connectivity distributes across a simple, AC-based segmentation of the putamen that is compatible with clinical workflows, and to examine whether this organization can be used to support presurgical planning for convection-enhanced drug delivery (CED) into a motor-dominated segment of the putamen.

In our cohort, motor-associated cortical and subcortical regions showed higher connectivity strength to the superior posterior putamen segment, while non-motor regions favored other segments. The motor-associated regions such as 3a/b, 4a/p, 6mp SMA, 6ma pre-SMA, the cerebellum, and the STN displayed a higher connectivity strength to the superior posterior segment of the putamen. Particularly, the 6mp SMA showed almost exclusive structural connectivity to this segment. Other regions, such as 3a/b, were more generally connected to both posterior segments, while the cerebellum and the STN were primarily linked to both superior segments of the putamen. Non-motor-associated regions exhibited different patterns. In particular, the insula region connects predominantly to the superior anterior segment and the amygdala almost entirely to the posterior inferior segment. Based on this pattern, we selected the superior posterior segment as the CED target. Using stereotactic planning and a simple infusion simulation, both frontal and occipital drug injection trajectories could be planned safely in most hemispheres (85% vs. 82.5% safe; mid-p McNemar *p* = 0.8462) and achieved comparable target-segment coverage, with no statistically significant differences between them (7.69% vs. 8.74%; paired *t*-test *p* = 0.4875). The findings were obtained using deterministic tractography implemented in CE-marked clinical software and indicate that even a coarse, AC-based segmentation can distinguish a superior posterior segment with predominantly motor-associated connectivity from other segments with more limbic or paralimbic connectivity.

The tractography and simulations presented here are intended as a planning adjunct. They provide a reproducible way to select a motor-dominant putaminal segment using a simple stereotactic segmentation and compare candidate trajectories under explicit safety constraints using a transparent geometric coverage metric. A prospective validation would be required for claiming any clinical benefits. At the same time, MRI-based presurgical simulation tools in other neurosurgical settings have been evaluated prospectively with measurable clinical benefit. For example, in a randomized controlled study of MRI-based VR presurgical planning with Dextroscope in skull base tumor surgery, the VR-planned group had shorter operative time and postoperative length of stay, fewer intraoperative cerebrovascular injuries, and higher Karnofsky Performance Scores at discharge and at 6 months compared with conventional planning (Yang et al., 2009). This supports the premise that MRI-based planning tools can improve outcomes when validated.

Our findings are broadly consistent with previous functional and structural research suggesting that the putamen has distinct subregions with unique functional roles. For instance, previous studies show that the anterior putamen has stronger connectivity with limbic structures such as the nucleus accumbens and amygdala, whereas the posterior putamen is more connected to motor-related regions like the cerebellum and sensorimotor cortex (Zhang et al., 2016; Liu et al., 2018). Studies by Choi et al. (2012) using resting-state functional connectivity MRT (fcMRI) characterized this functional organization, which aligns with findings in primate studies. Additionally, Di Martino et al. (2008) analyzed the functional connectivity of basal ganglia circuitry in humans, highlighting distinct connections between striatal regions and cortical motor, cognitive, and affective regions, providing another *in vivo* evidence of a functional organization described in animals. More recent work used functional connectivity gradients and “gradientography” in a large healthy cohort to derive a multiscale subcortical atlas, including a dorsoposterior putamen subdivision (PUT-DP) with relatively high group-averaged Dice coefficients and stable coupling to sensorimotor cortical networks (Tian et al., 2020). In this context, our superior posterior segment is coarser and defined by stereotactic geometry rather than by gradients, but it occupies a similar dorsoposterior location and shows a comparable bias toward motor-associated connections, while other quadrants show stronger non-motor connectivity. Taken together, these comparisons suggest that an AC-based quadrant scheme, although simple, can approximate the location of putaminal territories described in more detailed functional and multimodal parcellations.

The choice of an AC-based segmentation was motivated by its compatibility with established stereotactic practice. The anterior commissure (AC), together with the posterior commissure (PC), is routinely used to establish the AC-PC line (Starr et al., 2006; Ostrem et al., 2007). Although newer techniques propose using alternative markers, such as the putamen itself, for deep brain stimulation (DBS) targeting (Thompson et al., 2017), the AC remains a reliable and easily identifiable structure across imaging modalities. Furthermore, algorithms have been developed for automatic AC detection, aiding segmentation and registration (Ardekani and Bachman, 2009), reinforcing the AC’s role in functional neurosurgery. While newer techniques may offer promising alternatives, particularly in research settings, the use of the AC-based division remains highly practical in clinical environments where time, resources, and the experience of neurosurgeons with emerging tools can be limited. In our data, this simple segmentation was sufficient to highlight a superior posterior region with predominantly motor-associated connectivity, which may be helpful when selecting a putaminal target for CED in PD.

The simulation algorithm was used to explore whether the superior posterior motor-dominant segment could be reached safely and with a quantifiable degree of simulated target-segment coverage via frontal and occipital injection trajectories. Trajectories were planned under a 2 mm safety margin relative to sulci, ventricles and vasculature. In most hemispheres, at least one safe trajectory could be identified for each entry type. In one patient with marked atrophy, no safe frontal trajectory could be planned, while an occipital trajectory remained feasible. This suggests that anatomical variability can lead to asymmetries in feasibility between entries, and that both options may need to be considered at the planning stage. Within each planned trajectory, a small number of spherical infusion volumes, constrained to remain within the superior posterior segment and respect safety margins, were positioned using a genetic algorithm to maximize overlap with the target. Under these conservative assumptions, frontal and occipital trajectories achieved similar target-segment coverage (<10% on average), with no significant difference between them. The relatively low coverage values should be interpreted in the context of the modelling choices. First, coverage was calculated with respect to the entire volume of the superior posterior segment, which includes tissue that may be difficult to reach with a single straight trajectory because of the curved and tapering shape of the segment. Second, infusion volumes were required to be fully contained within the segment and to observe safety margins, which reduces the number and size of spheres that can be placed along a given trajectory. Third, the simulations were limited to a small number of trajectories and spheres in order to remain close to what might be realistically implemented in a clinical setting. Finally, the diffusion of the infused agent was approximated as isotropic and spherical, without modelling tissue anisotropy or catheter-related backflow. Under these constraints, the <10% coverage values are better regarded as conservative estimates and as relative measures for comparing candidate trajectories, rather than as estimates of maximal achievable coverage. Higher coverage might be possible with additional entry points, longer in-segment path lengths, different catheter designs, or more detailed biophysical models, but such approaches were beyond the scope of this study.

Nevertheless, our simulation allowed us to refine injection site placement and volume along each trajectory to achieve optimal target coverage within safe anatomical limits. Using an adaptive approach, the algorithm could adjust placement configurations in response to coverage feedback, supporting the need for flexible trajectory planning when anatomical differences make one approach preferable. This approach could be useful in deciding which trajectory to choose for maximal possible coverage in cases where multiple trajectories are feasible.

Convection-enhanced delivery (CED) shows potential for conditions like PD, though it comes with risks, such as mass effect from infused drugs and neurotoxicity at high drug concentrations (Slevin et al., 2006; Taylor et al., 2013). A targeted approach focusing on motor-associated regions of the putamen could, in principle, help limit exposure of non-motor regions. However, whether this translates into better clinical outcomes or altered dosing requirements cannot be answered by the present simulations. Such questions require empirical studies that measure *in vivo* distribution and relate delivery metrics to outcomes. Nevertheless, such an approach aligns with recommendations in previous research on structural connectivity-guided interventions, such as DBS of globus pallidus internus, for improved outcomes in PD (da Silva et al., 2017). The simulations presented here illustrate one way in which connectivity information and basic geometric modelling can be combined to estimate, in advance, how different trajectories might perform with respect to a chosen putaminal segment.

Several extensions could be considered in future work. On the connectivity side, using higher-resolution diffusion imaging and models that account for crossing fibers could refine the estimation of putaminal connectivity patterns and test whether the posterior–superior motor bias observed here remains stable under different modelling assumptions. Normalized connectivity metrics, such as relative streamline densities adjusted for seed volume and total streamlines, could reduce the influence of ROI size and inter-individual differences in brain size. On the functional side, task-based or resting-state fMRI could be used to confirm that the structurally defined target segment participates in motor networks in individual patients. With regards to segmentation, separate cortical normalization could be implemented to further improve the alignment between the atlas and patient-specific anatomy, reducing the manual workload even further. Likewise, future studies with larger datasets might incorporate advanced techniques, such as deep learning, to better simulate drug distribution, although this approach requires significant data and computational resources. On the biophysical side, incorporating tissue anisotropy, regional permeability, or more complex infusion profiles (such as ramped-rate protocols) into the simulations could help relate modelled coverage more directly to *in vivo* drug distribution.

This study also has several limitations that must be addressed. The sample size was relatively small and limited to patients with advanced PD undergoing DBS work-up, which may restrict how broadly our findings may apply to a wider PD population or other patient groups. The diffusion protocol used a single b-value and standard clinical resolution, and deterministic, tensor-based tractography was applied without explicit modelling of crossing fibers, which may reduce sensitivity in regions with complex fiber architecture. The putamen segmentation was intentionally simple, based on AC-PC geometry rather than subject-specific functional gradients, and therefore aggregates finer subregions within each quadrant. Additionally, while our semi-automated cortical segmentation method reduced manual workload, it still required manual adjustments, introducing variability. The infusion simulations used a spherical, isotropic diffusion model and were not validated against empirical infusion data. Future studies that include measurement of *in vivo* distribution and clinical outcomes will be required to validate whether this planning approach improves delivery, safety, or patient-relevant endpoints.

## Conclusion

This study used an AC-based four-quadrant segmentation and deterministic, clinically available tractography to characterize connectivity patterns within the putamen in patients with Parkinson’s disease and to explore their use for CED planning. The superior posterior segment showed the highest connectivity strength to motor-associated cortical and subcortical regions and was selected as the CED target. Using stereotactic planning and a simple geometric infusion model, both frontal and occipital drug injection trajectories into this segment could be planned safely in most hemispheres and yielded similarly comparable simulated target-segment coverage, with anatomical variation occasionally favoring one approach over the other. These findings suggest that connectivity mapping, even when derived from a coarse, AC-based segmentation and deterministic tractography, can help identify a motor-dominated putaminal segment and compare candidate trajectories for CED into this region. Further work with larger samples, crossing-fiber models, multimodal connectivity, more realistic diffusion models, and validation of infusion patterns will be needed to refine this approach.

## Data Availability

The raw data supporting the conclusions of this article will be made available by the authors, without undue reservation.
